# Clinical Advances in Metabolic Dysfunction-Associated Steatotic Liver Disease: Context, Measurement, and Treatment †

**DOI:** 10.3390/jcm15218634

**Published:** 2026-11-05

**Authors:** Hirsh D. Trivedi

**Affiliations:** Department of Internal Medicine, Dell Medical School, The University of Texas at Austin, Austin, TX 78712, USA; hirsh.trivedi@austin.utexas.edu

## 1. Introduction

Metabolic dysfunction-associated steatotic liver disease (MASLD) is now the most common chronic liver disease in the world. Pooled estimates place global prevalence near 30%, and the number continues to climb [1]. The 2023 multisociety nomenclature replaced NAFLD with MASLD. It anchored the diagnosis in the presence of cardiometabolic risk factors rather than in the exclusion of other causes [2]. The new name did more than retire stigmatizing language. It placed the liver inside a systemic metabolic disease.

The clinical picture has shifted quickly. Two drugs now carry regulatory approval in the United States for noncirrhotic metabolic dysfunction-associated steatohepatitis (MASH) with moderate to advanced fibrosis. Resmetirom received accelerated approval in 2024 after the MAESTRO-NASH trial [3]. Semaglutide 2.4 mg followed in August 2025 based on part 1 of ESSENCE [4]. These approvals change the question at the front end of care. For two decades, the practical question was whether to look for fibrosis when few treatments existed. The question now is how to find the right patients, in sufficient numbers, with tools that work where patients are seen.

This Special Issue set out to collect work on novel concepts in MASLD diagnosis and management. The six published papers fall into three groups. Two examine the social and structural context in which MASLD develops. Three evaluate tools for measuring disease, from elastography to routine blood indices to markers of intestinal barrier function. One reviews the therapeutic landscape. Together, they map the distance between what is possible in a trial and what is achievable in a clinic.

## 2. The Context in Which MASLD Develops

Two studies in this issue examine education as a determinant of MASLD in very different populations.

Donghia et al. studied 2909 participants from the MICOL and NUTRIHEP cohorts in southern Italy [5]. They found a graded inverse association between educational attainment and MASLD. Compared with the lowest education group, the odds ratios for MASLD were 0.50 for middle school, 0.29 for high school, and 0.24 for university graduates. The gradient was steep and consistent. The authors argue that education policy may be a lever on liver disease, a point rarely made in hepatology journals.

Singh et al. asked a related question in a nationally representative US sample [6]. They used NHANES 2017 to 2018 and defined MASLD by controlled attenuation parameter (CAP). Higher education was associated with lower odds of MASLD after full adjustment (OR 0.77, 95% CI 0.62 to 0.97). Higher income was not associated with MASLD (OR 1.12, 95% CI 0.91 to 1.37). The more important finding was an interaction. Education was protective among participants with food security (OR 0.71, 95% CI 0.55 to 0.91). It showed no protective association among those with food insecurity (OR 1.26, 95% CI 0.76 to 2.11).

Read together, these studies suggest that education matters and that its benefit depends on the conditions around it. Knowledge about nutrition cannot change a diet when reliable access to food is absent. This is consistent with earlier NHANES work showing that food insecurity is independently associated with higher all-cause mortality in adults with steatotic liver disease and advanced fibrosis [7]. It also fits a growing framework that treats food insecurity as an upstream and modifiable risk factor for MASLD [8].

Both studies are cross-sectional. Neither can establish causation, and residual confounding by occupation, neighborhood, and access to care is likely. Still, they move attention away from individual behavior and toward the structures that shape metabolic risk. They also suggest a practical step. Food insecurity screening is brief, validated, and actionable. It belongs in metabolic liver clinics alongside FIB-4.

## 3. Measuring Disease with the Tools at Hand

Noninvasive tests have largely replaced liver biopsy for risk stratification. Current guidance supports a sequential approach. FIB-4 is used first, often in primary care. Elastography or a specialized blood test follows when FIB-4 is indeterminate or elevated [9,10]. That pathway assumes the second-tier test is available and accurate. Three papers in this issue test that assumption from different angles.

### 3.1. Elastography and the Problem of Severe Steatosis

Losurdo et al. compared vibration-controlled transient elastography (FibroScan) with point shear wave elastography (pSWE) in 85 patients on the same day [11]. Overall correlation was substantial (r = 0.66). Correlation rose as steatosis increased from S0 to S2 (r = 0.79, 0.81, and 0.85). It then fell sharply in S3 steatosis (r = 0.48), with poor agreement.

This matters for two reasons. First, pSWE runs on conventional ultrasound platforms that many centers already own. It can widen access to fibrosis assessment where dedicated transient elastography is not funded. The data support treating the two methods as interchangeable in mild to moderate steatosis. Second, severe steatosis is common in exactly the patients being referred. Prior work has shown that liver stiffness can overestimate fibrosis in steatotic liver disease and that accounting for CAP improves interpretation [12]. When steatosis is severe and the two methods disagree, a third modality such as magnetic resonance elastography or the ELF test is reasonable before a treatment decision. The study is small and lacks a histologic reference standard. Confirmation in a biopsy-anchored cohort is the next step.

### 3.2. A Routine Blood Index with an Unexpected Direction

Kirik et al. evaluated the blood urea nitrogen to serum albumin ratio (BAR) in 425 patients with biopsy-proven MASLD from the Turkish MASLD Biobank [13]. BAR was lower in patients with significant fibrosis and in those with MASH. It declined stepwise from F0 to F4. It remained independently associated with significant fibrosis after adjustment (OR 0.813). The authors are candid that discrimination was poor, with an AUROC of 0.586. BAR is not a diagnostic test.

The direction of the signal is the interesting part. In other conditions, a higher BAR tracks with systemic inflammation. Here the relationship is inverted. One plausible explanation is reduced hepatic urea synthesis, which has been described in steatotic liver disease and may precede overt functional decline [14]. If so, BAR may be a crude window onto hepatocellular metabolic capacity rather than inflammation. That hypothesis is testable, and well-characterized biopsy cohorts like this one are the place to test it.

### 3.3. The Gut-Liver Axis in Children

Wierzbicka-Rucińska et al. studied 60 children with MASLD and overweight or obesity, 33 children with overweight or obesity without MASLD, and 16 healthy controls [15]. They measured alpha-defensins 5 and 6 as markers of Paneth cell antimicrobial activity. They also measured intestinal and liver fatty acid-binding proteins (I-FABP and L-FABP) as markers of epithelial injury. L-FABP was highest in children with MASLD. Defensin 6 was lowest in children with obesity alone and higher in those with MASLD. Defensin 5 did not differ. Lipid profiles also did not differ between groups. That suggests these markers capture biology that conventional metabolic laboratory tests miss.

The work extends the gut-liver axis model [16] into pediatrics, where early mechanistic insight has the longest runway. MASLD was defined by ultrasound and alanine aminotransferase rather than histology, and the control group was small. For now, these markers are research tools.

## 4. The Treatment Landscape

Patel reviews current and emerging therapy for MASLD [17]. The review frames lifestyle intervention as foundational. It then surveys glucagon-like peptide-1 (GLP-1) receptor agonists, resmetirom, the pan-PPAR agonist lanifibranor, and fibroblast growth factor 21 (FGF21) analogs.

The evidence has continued to move. In MAESTRO-NASH, resmetirom achieved both MASH resolution and fibrosis improvement over placebo at 52 weeks [3]. In part 1 of ESSENCE, semaglutide 2.4 mg resolved steatohepatitis without worsening fibrosis in 62.9% of patients versus 34.3% with placebo. Fibrosis improved without worsening steatohepatitis in 36.8% versus 22.4% [4]. In compensated cirrhosis, the FGF21 analog efruxifermin did not meet its 36-week primary endpoint in the phase 2b SYMMETRY trial. At 96 weeks, however, the 50 mg dose showed fibrosis regression over placebo [18]. If confirmed in phase 3, that would be the first evidence that pharmacotherapy can reverse MASH cirrhosis.

Three gaps remain. Current approvals rest on histologic surrogates, and clinical outcome data are still pending. Cirrhosis, where the risk of liver events is highest, has no approved therapy, and no drug helps a patient who is never identified. The bottleneck has moved from the pharmacy to the front door.

## 5. What This Special Issue Adds and What Comes Next

The papers in this issue point toward four priorities.

First, risk assessment should include social context. Food security and educational opportunity shape who develops MASLD and how much benefit counseling can deliver. Screening for food insecurity is inexpensive. It can connect patients to resources that a prescription cannot replace.

Second, noninvasive tests must be validated in the patients who will actually be tested. Severe steatosis, type 2 diabetes, and pediatric age each change test performance. Validation across diverse ancestries and care settings remains incomplete.

Third, inexpensive markers deserve rigorous testing, including honest reporting of modest results. A modest AUROC published with appropriate caveats is more useful to the field than an overfit score.

Fourth, therapy is no longer the limiting step; case finding is. Most people with MASLD are seen in primary care, endocrinology, and cardiology clinics. Primary care teams already carry a heavy preventive workload with limited dedicated time. Reimbursement models rarely support fibrosis assessment or sustained metabolic care. Funding elastography in community settings, building clear referral pathways, and supporting shared care between primary care and hepatology will decide whether new therapies reach the patients who need them.

## 6. Conclusions

MASLD is a systemic disease. It develops within social and structural conditions, progresses silently, and now has treatments. The contributions in this Special Issue address each of those facts. I thank the authors for their work, the reviewers for their time, and the editorial staff of the *Journal of Clinical Medicine* for their support.

## References

[1] Younossi Z.M., Golabi P., Paik J.M., Henry A., Van Dongen C., Henry L. (2023). The global epidemiology of nonalcoholic fatty liver disease (NAFLD) and nonalcoholic steatohepatitis (NASH): A systematic review. Hepatology.

[2] NAFLD Nomenclature Consensus Group (2023). A multisociety Delphi consensus statement on new fatty liver disease nomenclature. J. Hepatol..

[3] Harrison S.A., Bedossa P., Guy C.D., Schattenberg J.M., Loomba R., Taub R., Dominic L., Moussa S.E., Neff G.W., Rinella M.E. (2024). A Phase 3, Randomized, Controlled Trial of Resmetirom in NASH with Liver Fibrosis. N. Engl. J. Med..

[4] Sanyal A.J., Newsome P.N., Kliers I., Østergaard L.H., Long M.T., Kjær M.S., Cali A.M.G., Bugianesi E., Rinella M.E., Roden M. (2025). Phase 3 Trial of Semaglutide in Metabolic Dysfunction-Associated Steatohepatitis. N. Engl. J. Med..

[5] Donghia R., Bonfiglio C., Giannelli G., Tatoli R. (2025). Impact of Education on Metabolic Dysfunction-Associated Steatotic Liver Disease (MASLD): A Southern Italy Cohort-Based Study. J. Clin. Med..

[6] Singh V., Cheng S., Velazquez A., Trivedi H.D., Kwan A.C. (2025). The Impact of Social Determinants of Health on Metabolic Dysfunction-Associated Steatotic Liver Disease Among Adults in the United States. J. Clin. Med..

[7] Kardashian A., Dodge J.L., Terrault N.A. (2022). Food Insecurity is Associated with Mortality Among U.S. Adults with Nonalcoholic Fatty Liver Disease and Advanced Fibrosis. Clin. Gastroenterol. Hepatol..

[8] Ivancovsky-Wajcman D., Carrieri P., Younossi Z.M., Pericàs J.M., Zelber-Sagi S., Lazarus J.V. (2024). Food inequity and insecurity and MASLD: Burden, challenges, and interventions. Nat. Rev. Gastroenterol. Hepatol..

[9] European Association for the Study of the Liver (EASL), European Association for the Study of Diabetes (EASD), European Association for the Study of Obesity (EASO) (2024). EASL-EASD-EASO Clinical Practice Guidelines on the management of metabolic dysfunction-associated steatotic liver disease (MASLD). J. Hepatol..

[10] Rinella M.E., Neuschwander-Tetri B.A., Siddiqui M.S., Abdelmalek M.F., Caldwell S., Barb D., Kleiner D.E., Loomba R. (2023). AASLD Practice Guidance on the clinical assessment and management of nonalcoholic fatty liver disease. Hepatology.

[11] Losurdo G., Castellaneta A., Di Nuccio C., Dell’Aquila P., Ditonno I., Novielli D., Continisio A., De Bellis M., Di Leo A., Principi M. (2025). Comparison Between Transient Elastography and Point Shear Wave Elastography in the Assessment of Liver Fibrosis According to the Grade of Liver Steatosis. J. Clin. Med..

[12] Petta S., Wong V.W.-S., Cammà C., Hiriart J.-B., Wong G.L.-H., Marra F., Vergniol J., Wing-Huan Chan A., Di Marco V., Merrouche W. (2017). Improved noninvasive prediction of liver fibrosis by liver stiffness measurement in patients with nonalcoholic fatty liver disease accounting for controlled attenuation parameter values. Hepatology.

[13] Kirik A., Kaya E., Keklikkiran C., Yilmaz Y. (2026). Blood Urea Nitrogen to Serum Albumin Ratio Is Associated with MASH and Significant Fibrosis: Report from a Turkish Biopsy-Proven MASLD Cohort. J. Clin. Med..

[14] De Chiara F., Heebøll S., Marrone G., Montoliu C., Hamilton-Dutoit S., Ferrandez A., Andreola F., Rombours K., Grønbæk H., Felipo V. (2018). Urea cycle dysregulation in non-alcoholic fatty liver disease. J. Hepatol..

[15] Wierzbicka-Rucińska A., Konopka E., Więckowski S., Jańczyk W., Świąder-Leśniak A., Świderska J., Trojanek J., Kułaga Z., Socha P., Bierła J. (2025). Evaluation of Defensins as Markers of Gut Microbiota Disturbances in Children with Obesity and Metabolic Dysfunction-Associated Steatotic Liver Disease (MASLD). J. Clin. Med..

[16] Albillos A., de Gottardi A., Rescigno M. (2020). The gut-liver axis in liver disease: Pathophysiological basis for therapy. J. Hepatol..

[17] Patel P. (2025). Treatment Landscape of Metabolic-Dysfunction-Associated Steatotic Liver Disease. J. Clin. Med..

[18] Noureddin M., Rinella M.E., Chalasani N.P., Neff G.W., Lucas K.J., Rodriguez M.E., Rudraraju M., Patil R., Behling C., Burch M. (2025). Efruxifermin in Compensated Liver Cirrhosis Caused by MASH. N. Engl. J. Med..

